# An exploration of cultural beliefs and practices across the Southern Ground-Hornbill’s range in Africa

**DOI:** 10.1186/1746-4269-10-28

**Published:** 2014-03-26

**Authors:** Hendri Coetzee, Werner Nell, Leon van Rensburg

**Affiliations:** 1 Research Unit for Environmental Sciences & Management, North-West University, Potchefstroom, South Africa; 2 School of Behavioural Sciences, North-West University, Vaal Triangle Campus, South Africa

**Keywords:** Southern Ground-Hornbill, *Bucorvus leadbeateri*, Cultural beliefs, Cultural practices, Intervention programme, Surrogate species, Conservation

## Abstract

**Background:**

This article explores cultural beliefs and practices related to the Southern Ground-Hornbill (SGH) (*Bucorvus leadbeateri*) in nine southern and east-African countries.

**Methods:**

A qualitative, thematic content-analysis approach was followed. Ninety-eight participants took part in the study. Interviews and group discussions were used as the main data gathering methods. Each interview was digitally recorded and transcribed. Data were analysed by means of thematic content analysis.

**Results:**

The main themes that emerged from the data analysis indicated that beliefs and practices relate to the SGH as being (a) a bringer or signifier of death/destruction/loss/ deprivation, with the bird commonly being regarded as a bad omen of evil spirits and announcer of calamities; (b) a protector against evil spirits, against lightning and against drought; (c) an enabler/causer of altered perceptions, which include remote viewing, foreseeing the future, and creating an illusion; and (d) a timekeeper that announces the beginning and end of a working day and of seasonal changes.

**Conclusions:**

Knowledge about the use of the SGH in cultural practices can contribute to conservation efforts in at least two significant ways: Firstly, beliefs and practices that were identified in this study as having potentially protective consequences for the SGH can now be specifically targeted and strengthened in future interventions. Secondly, destructive beliefs and practices that were identified can now be changed by means of the implementation of an intervention programme in countries where it is needed.

## Background

Birds play a significant role in the lives of people across virtually all cultures and continents [1]. In most cultures, significant beliefs have developed in relation to birds, usually as a result of direct and regular contact with specific bird species, especially in cases where such birds possess prominent visual, auditory or behavioural characteristics [2]. In turn, these beliefs, which are usually constructed through a process of social interaction [3], give rise to a variety of practices and behaviours that centre around these bird species. These practices and behaviours range from actual uses, such as hunting and eating certain bird species [4], to using birds in cultural practices, such as rituals, ceremonial acts [5], and the making of traditional medicines [6], and also making use of birds for more symbolic purposes as is the case in art such as painting, dancing, or sculpture [7]. The consequences of these beliefs could vary from being inimical and destructive to the species, to being of no consequence or even to having a protective influence on the conservation of the species. For example, the use of animal parts in traditional medicine often necessitates that the bird be killed, which exerts a destructive influence as far as species conservation is concerned [8]. Conversely, traditional beliefs may also lead to the protection of certain species by specific cultures in the form of food taboos [9].

**Figure 1:**
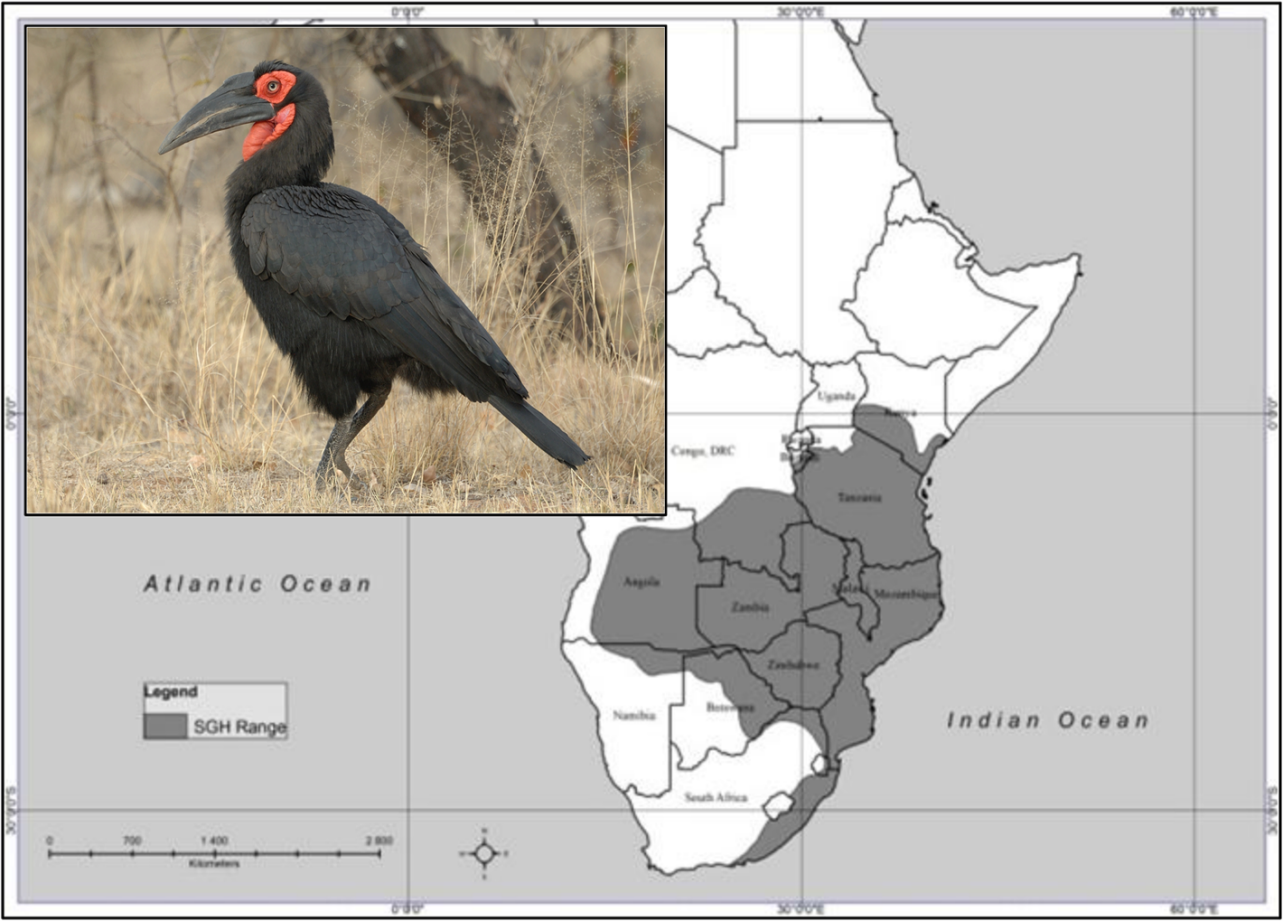
Distribution and photograph of an adult SGH male.

The Southern Ground-Hornbill (SGH) (*Bucorvus leadbeateri*) is the epitome of a species that has been in regular contact with many different cultures over a prolonged period of time, and that has all the characteristics that could result in the development of a large number of cultural beliefs and practices. Figure 1 provides an indication of the SGH and its fairly extensive distributional range.

The bird historically occurred in all southern and east-African countries south of the equator from the southern parts of Kenya, all the way down to South Africa and west into Uganda, Rwanda, Burundi, the Democratic Republic of the Congo, and Angola [10]. It is known for its deep, four-note booming call and its large size (90 cm in height, and weighing between 4–6 kg). It is also one of only a few birds with a combination of aposematic colours (black, white and red) that are found in African savannas. All of these factors make the SGH very conspicuous, especially in the open grassveld and savanna-woodland habitats where it often occurs in pairs of cooperatively-breeding groups of up to a dozen birds (average 3.5) [11]. It spends most of its time foraging on the ground for small animals (up to the size of hares) in large territories with densities of 1 group per 100–250 km^2^[11,12].

The species’ conservation status is affected by biological factors which include a long mean lifespan (estimated 50–60 years), low reproductive rate (one chick fledged per group every nine years on average), and long age to sexual maturity (4–6 years) and first breeding (>10 years). In addition to these biological factors, the species has now disappeared from large parts of its historical range and is mainly restricted to protected areas, probably as a result of such anthropogenic threats as habitat destruction, direct persecution, indirect poisoning, electrocution, trade in live specimens, and use in traditional cultural practices [13]. Of all these threats, the least studied is the SGH’s use in cultural practices.

A number of hornbill species are used in cultural practices, particularly in Asia [10,14]. The SGH is no exception. In the case of the SGH, most documented knowledge related to the bird’s role in cultural beliefs and practices stems from the southern parts of its range where perceptions of the bird relate to the self [15], to important others in people’s social and spiritual worlds [16-20] and to the environment, including beliefs and practices pertaining to hunting and food [16,18] and to rain, lightning and drought [17,19,21-23]. However, only a small number of studies have recorded beliefs and practices in the northern parts of the SGH’s range, most notably in Tanzania [24] and Kenya [2] where these beliefs also relate to their social and spiritual worlds.

However, a major gap exists in documented knowledge about the beliefs and practices related to the SGH’s use in Rwanda, Burundi, Zambia, the Democratic Republic of the Congo, Angola, Namibia, and Botswana. Furthermore, most of the published records on this topic seem outdated and/or anecdotal. From an ethno-ornithological perspective, it is important to document the beliefs and practices related to the SGH, not only because it forms part of the African cultural heritage but also because such knowledge could potentially contribute to conservation efforts in a variety of different ways as a result of an enhanced understanding of the dynamics of the human – SGH relationship. Consequently, this study set out to address the following research questions:

• What are the cultural beliefs and practices associated with the SGH throughout its range in eastern and southern Africa?

• What are the causes and consequences of these beliefs and practices?

## Method

A qualitative, thematic content-analysis approach [25] was deemed to be the most appropriate research strategy as the aim of the study was to obtain a culturally sensitive, insider perspective on the beliefs and practices related to the SGH. This approach is based on an interpretivist paradigm, which also serves as the philosophical and theoretical underpinning of this study. Interpretive research is based on knowledge obtained through social constructs such as language, consciousness, shared meanings, document tools, and other artefacts [26]. A qualitative approach is ideally suited in instances when researchers wish to enter the life-world of the participants and view phenomena from their perspective, as was the case with this study.

### Participants

Ninety-eight participants took part in the study. They were selected by means of purposive sampling, a strategy which involves the selection of individuals who are familiar with a particular context and topic and who are best able to provide the researchers with the information required to answer the research questions [27]. Of these participants, five were from Burundi, seven from Rwanda, eight from Uganda, 14 from Kenya, three from Tanzania, six from Malawi, seven from Zambia, six from Zimbabwe, seven from Mozambique and 35 from South Africa. Their occupations and roles in each country ranged from local traditional, political, and religious leaders (n = 6); elders and traditional healers in local communities (n = 37); other informed local community members (n = 22); park wardens (n = 11), technical staff, guides and trackers in national parks and game reserves (n = 4); local conservation and bird experts from NGOs such as Birdlife, the World Wildlife Fund and the various Nature branches (n = 8); to academic staff from universities and museums (n = 10).

### Procedure

In South Africa, initial fieldwork commenced in 2011 at Faraday traditional medicine market in Johannesburg (Gauteng), which was followed by more in-depth research at several traditional medicine markets throughout the Eastern Cape Province and KwaZulu-Natal towards the end of 2011. This was followed by even more extensive work in the western parts of Zimbabwe and in the region of Maputo in Mozambique in 2012. Fieldwork was then extended to Rwanda, Uganda, Kenya, Tanzania, Zambia and Malawi during 2013. Finally, a local ornithology expert from Rwanda was used to do follow-up work in Burundi.

Entry into each country was negotiated through local conservation and/or bird experts at universities and museums in all the countries. These participants were all informed about the aim of the project and, in addition to providing information, in some instances also acted as guides and translators during the fieldwork phase of the study.

Great care was taken to ensure that the study was conducted in an ethical manner. Participants were all informed of the nature of the study, as well as of what their participation would entail, and of their right to withdraw during any stage of the research without having to give reasons for doing so. It was also explained to participants that all information shared with the researchers would be treated with confidentiality, especially in relation to the use of the SGH which is a protected species in many countries. Following this, full informed consent was obtained from all participants before proceeding with the interviews.

Data were collected by means of semi-structured, face-to-face interviews [27] as well as electronic correspondence with participants. During the interviews, a colour photograph of an SGH (Figure 1) was shown to participants as a prelude to asking them the following questions: a) ‘Do you know this bird?’, and if so, (b) ‘Do you know anything about its use in cultural practices?’ Depending on the answers that were given, a number of follow-up questions were asked to further explore and clarify the participants’ views. All interviews were conducted according to this procedure. Occasionally the interviews took the form of group discussions, where a similar approach was followed. Interviews ranged from 17 minutes to nearly two hours. Both the interviews and focus group discussions were digitally recorded with full permission of the participants and transcribed verbatim after each data-gathering session.

In line with the standard practiced used in qualitative research, data were collected until a point of saturation was reached. This involves seeking and interviewing new participants until no new themes emerged related to the SGH’s use in cultural practices [27].

The process of data analysis was aimed at identifying specific concepts in the transcripts that elucidated the use of SGHs in cultural practices. As prescribed by Corbin and Strauss [28], data were initially subjected to a process of open coding, during which descriptive codes were assigned to fragments of text. Following this, codes were inductively grouped together into categories based on conceptual similarities as part of a selective coding procedure. Finally, each category and theme was studied in detail again to make sure the original data truly supports these categories.

## Results

The themes and categories that emerged from the qualitative analysis of the data are reported in Table 1. Main themes are reported in the first column, followed by the categories in the second column, and verbatim quotes from the participants (to support each theme and category) in the third column. The remainder of this section is devoted to a discussion of these themes and categories. Each theme and category is discussed according to country (from south to north, with the exception of Rwanda and Uganda where the SGH no longer occurs in significant numbers). These discussions start with an outline of the causes underlying the use of the SGH, followed by an overview of beliefs related to the SGH, a discussion of the specific use/s thereof (i.e. practices) and, finally, of the consequences of these practices for both the user and the bird.

**Table 1:** Themes and categories related to beliefs and practices associated with the SGH

**Theme**	**Category**	**Verbatim examples**
**Bringer/signifier of death/ destruction/ loss/ deprivation**	Bad omen of evil spirits	*“The SGH is the carrier of dead souls… and particularly those of angry, avenging or ancestral spirits.”*
	Announcing calamities (death and destruction)	*“An SGH perched on top of a person’s roof, or in or near a community’s village, is a sign of death…”*
**Protective influence**	Protection against evil spirits	*“I ground it up, burn and use the ash which I rubbed into incisions in my joints. It makes me strong to withstand the onslaughts of evil spirits.”*
	Protection against lightning	*“We use it to protect our property against lightning.”*
	Protection against food shortages	“*When we use the bird we know that rain will follow*”
**Enables or causes altered perceptions**	Remote viewing	“*The bird is a predictor. It can stand here and it will know what is happening in Soweto* (a nearby township where most market-goers reside).”
	Foreseeing the future	“*The bird helps us to find food, like honey and small antelope*”
	Creating an illusion	“*We use it to increase the weight of our harvested crops*”
**Timekeeper**	Working day	“*It tells us when to wake up or go to bed.*”
	Change in season	*“If we see that bird, it means that it is going to rain…this means we must prepare our field to plant our crops.”*

### A bringer/signifier of death/destruction/loss/deprivation

One of the primary themes that emerged from the data was that the SGH is often perceived as a bringer or signifier of death/destruction/loss/deprivation. More specifically, it is believed that seeing or encountering this bird in the wild, or having it enter or approach a village or homestead signifies impending calamity, which most often was believed to involve the death of someone known to the individual, or the damage and destruction of personal property. These beliefs are particularly prevalent in South Africa, Zimbabwe and Malawi, where participants believed that “*when a SGH lands on a person’s roof something bad is going to happen*” and that “*it is a sign of death, especially when encountered in odd numbers* (e.g. 1, 3, 5, etc.)”. Variations on this belief occurred in Burundi, parts of Kenya, Tanzania, Zambia and Mozambique, where participants believed that “*the SGH is an unlucky and an aggressive bird associated with evil and death*”. This also confirms earlier reports by Wilfred [24] who found that in Tanzania, the SGH is often perceived as the carrier of dead souls and particularly those of angry, avenging or ancestral spirits.

The consequences of these beliefs appear to be beneficial in parts of Kenya and in cases where people believe that the bird should be avoided or not be killed, as reflected in the words of one participant who said that “*the bird should not be killed, because it has the power to bring death and destruction*”. Likewise, in Zimbabwe, it is forbidden to chase, kill or even pick up a feather of an SGH, which should, according to locals, just be ignored and allowed to blow away in the wind because it could bring misfortune to an individual or village [23]. However, in these beliefs could possibly be destructive in cases where the bird is chased, stoned or killed as a result of its association with death or other bad omens (for example in Zambia and parts of Kenya).

### A protective influence

The second most prominent theme that emerged from the data was that the SGH is often perceived as a protective influence, most especially against evil spirits, lightning and drought. In many African cultures, lightning and drought are often perceived to be a manifestation of witchcraft or punishment from their ancestors [29]. These beliefs are prevalent across the bird’s range, where participants believed that “*an SGH can be used as protection against evil spirits and witchcraft*”, “*an SGH can be used to protect a person or property against lightning*” and that “*the bird can be used to bring rain*”. In the case of evil spirits, this “protection” does not refer to physical protection as such, but rather to the creation of a strong personality that would be able to withstand the attacks of such evil spirits. To harness the protective powers believed to be contained in the SGH, parts of the SGH are typically removed from a bird that was either specifically killed for this purpose or found dead in the surrounding areas. The practices to protect oneself or one’s property (e.g. homestead, crops and other belongings) against lightning involve mixing various parts of the SGH (e.g. feathers and feet) with plant parts and animal fat, and smearing this mixture on various parts of the homestead. This topic has also been explored in detail by Koopman [30], who confirmed that in the instances where parts of the SGH were used to protect a person against being struck by lightning, the traditional medicine is rubbed on strategic points around the homestead.

The finding that the SGH is often used for protection against drought also confirms two other documented examples from South Africa and Mozambique [17,20], where parts of the SGH are used to bring rain in times of severe drought. The ritual involves dancing and singing special songs related to the bird, and using parts of or a whole bird that is normally suspended from a branch overhanging a river or tied with a rope in a dry riverbed. The bird is also guarded by members of the community, so that it can be removed when enough rain has fallen. The results of this study as well as the aforementioned two references confirm earlier reports by Vernon [17] who documented one such ritual in detail.

The consequences of these beliefs appear to be neutral in countries such as Malawi and Zambia where the bird is not used for any form of protection and generally ignored and left alone, but they are definitely destructive in instances where birds are killed for use in rituals to protect against lightning or to bring rain, such as is the case in South Africa.

### Enables/causes altered perceptions (e.g. a different mode of seeing)

The third most prominent theme that emerged from the data was that the SGH is often perceived as an enabler of altered perceptions. In this capacity, it is believed that the SGH has the power to cause either the user, or others who view the user to perceive reality in an expanded or altered way. On the one hand, the SGH is used to alter reality by causing illusions or by distorting perceptions, and on the other hand it is employed to create an expanded state of awareness which usually involves the capacity of remote viewing of realities that are occurring beyond the current location of the user. These beliefs are particularly prevalent in Zimbabwe and Malawi where participants believed that “*the SGH can be used to foresee and avoid an enemy or unwelcome guest like a debt collector*” and that “*the SGH can be used to create an illusion of harvested crops being heavier that they actually are*” and “*the bird can be used to find food such as honey and small antelopes*”. The latter also confirms and are further supported by Maasdorp’s study [16] in Zimbabwe, where she recorded several beliefs such as that “*the SGH must have special powers to find mice and tortoises, because it does not have the high vantage point that vultures have to look down on carcases in flight*” and “*the SGH must be a prophet as it can predict where to find mice, dig them up, and eat them*”.

Participants said that when they want to harness the bird’s power in this particular way, they take the ashes of the bird and put it under their tongue, or sniff up a small amount before going to sleep.

In South Africa, the bird is also used to give authority to local leaders, thereby causing such individuals to be perceived differently by members of local communities. This strategy involves leaders using the bird to give themselves a deep voice which, according to their beliefs, shows authority. To harness the bird’s powers in this way involves a ritual where the head of the bird is placed in the leader’s bath water before he bathes.

The consequences of these beliefs for the SGH appear to be mostly destructive, because in all cases, it involves the capturing and killing of actual birds in order to obtain the parts necessary to perform these rituals.

### Timekeeper

The final theme that emerged from the data was that the SGH is often perceived as a timekeeper in relation to daily and seasonal changes. In the first case, two variations were encountered, where the appearance of SGHs is either perceived as a signal of a change from the wet to the dry season in the northern parts of its range, or from dry to wet in the southern parts of its range. These beliefs seem to correspond with the bird’s seasonal movements in parts of their range, and are particularly prevalent in South Africa, where participants believed that “*when an SGH calls, it is the start of their rain season*”. Variations on this belief occurred in the countries Kenya and Tanzania where participants believed that if they see an SGH, “*it is the start of their dry season and that they should move their cattle*”. In other parts of the bird’s range, comparable beliefs were found. For example, in Malawi it is believed that “*when they see or hear the bird, they must start preparing their fields*” and in Zambia “*that it won’t rain while the SGH is still in its nest”*’.

In their second role as a daily timekeeper, the call of the bird is used in the southern parts of Tanzania to announce the start and end of a working day, especially during certain times of the year, when dense cloud cover often makes it hard to discern the beginning and ending of the day.

The consequences of these beliefs appear to be neutral or even beneficial in cases where the SGH is not physically used in cultural practices, for example where its call is an indication of the beginning or end of a working day, but they could possibly be mildly destructive where the bird is stoned or flushed from its nest or even killed when people do not want the rain to end, or when they want the rain to start.

## Discussion

When considering the overall context in which the SGH is used, it appears that the SGH is most often used by local communities in Kenya, Tanzania, Malawi, Zambia, Zimbabwe, South Africa and Mozambique, all of which coincide with the SGH’s distributional range. (However, use of the SGH in the Democratic Republic of the Congo, Angola, Northern Namibia, and Botswana could not be confirmed). Within these contexts, certain causal conditions are encountered that act as stimuli for the SGH’s use in cultural practices. Such conditions typically revolve around attempts to deal with existential uncertainties resulting from natural phenomena such as droughts and lightning, or from an uncertain and unpredictable future. It would appear that the SGH’s distinctive coloration, call, and behaviour played a significant role in causing it to be selected as a culturally significant species that is used in direct, indirect, and symbolic ways to deal with these uncertainties. Using the bird in these practices empowers local users by helping them to survive and cope with natural phenomena over which they normally do not have any power. Most importantly, though, it helps them deal with such strong underlying emotions as fear, uncertainty and insecurity by giving them a sense of control. It also reflects the keen observation of the seasonal movements of the SGH which enables them to predict the change in seasons, which facilitates the timing of important activities, such as when to plant crops, or move their cattle to greener pastures.

On the other hand, cultural use of the SGH also puts pressure on the bird’s continued survival, especially in the light of its current threatened status. The specific ways in which the SGH is used in cultural practices, and the reasons informing these uses, have important implications for future interventions. Specifically, an understanding of these aspects can help with the identification of factors that may have an influence on the SGH’s future use, and thereby serve to lay the foundation for the development of culturally sensitive intervention programmes aimed at conserving the SGH.

This is especially pertinent in light of the observation that there seems to be a general lack of intervention programmes aimed at reducing the impact of cultural practices on threatened species. In the case of the SGH, this study laid the foundation for the development of an intervention programme, firstly through the identification of destructive beliefs and practices which could be directly targeted by intervention strategies, and secondly by identifying potentially protective practices which could be reinforced in order to facilitate the conservation of the SGH in a culturally sensitive manner.

## Conclusion

This study explored the most significant cultural beliefs and practices in relation to the SGH in nine southern and east-African countries across the bird’s range. Four main themes emerged from the data, and indicated that the SGH was primarily viewed as an omen or signifier of death, loss and destruction, as a protective influence, as an enabler of altered perceptions and remote viewing, and as a timekeeper in relation to seasonal and daily changes. Knowledge about SGH use in cultural practices can contribute to conservation efforts in at least two significant ways: First, the possibility now exists to create interventions aimed at strengthening those beliefs and practices that have protective consequences for the SGH, and second, destructive beliefs and practices that were identified can now be targeted effectively by means of the implementation of focused intervention programmes in countries where the SGH is under threat as a result of destructive cultural beliefs and practices.

## Competing interests

The authors declare that they have no competing interests.

## Authors’ contributions

All authors conceptualised the study. HC and WN carried out the fieldwork and wrote the manuscript. All authors read and approved the final manuscript.
